# Association Between Oral Behaviors and Symptoms of Anxiety and Depression in Romanian Adults Attending Private Dental Practices: A Cross-Sectional Observational Study

**DOI:** 10.3390/jcm15114207

**Published:** 2026-05-29

**Authors:** Alexandra Lavinia Vlad, Ioana Scrobota, Ioan Andrei Țig, Raluca Ortensia Cristina Iurcov, Anca Maria Fratila, Gabriela Ciavoi

**Affiliations:** 1 Doctoral School of Biomedical Sciences, Faculty of Medicine and Pharmacy, University of Oradea, 410087 Oradea, Romania; alvlad@uoradea.ro; 2 Department of Dental Medicine, Faculty of Medicine and Pharmacy, University of Oradea, 410068 Oradea, Romania; ioana_scrobota@uoradea.ro (I.S.); gciavoi@uoradea.ro (G.C.); 3 Faculty of Medicine, Lucian Blaga University of Sibiu, 550169 Sibiu, Romania; anca.fratila@ulbsibiu.ro; 4 Military Clinical Emergency Hospital of Sibiu, 550024 Sibiu, Romania

**Keywords:** oral behaviors, Oral Behaviors Checklist (OBC-21), anxiety, depression, GAD-7, PHQ-9, temporomandibular disorders (TMD), biopsychosocial model

## Abstract

**Background/Objectives:** Oral behaviors are increasingly considered relevant within the biopsychosocial framework of temporomandibular disorders, yet their relationship with emotional symptoms remains insufficiently characterized in general adult populations. This study investigated the association between the frequency of oral behaviors and the severity of anxiety and depression symptoms in adults. **Methods:** This observational, cross-sectional, multicenter study included 460 adults recruited from private dental practices. Oral behaviors were assessed using the Oral Behaviors Checklist (OBC-21), while anxiety and depression were evaluated using Generalized Anxiety Disorder-7 (GAD-7) and Patient Health Questionnaire-9 (PHQ-9). Associations were examined using Spearman correlations and generalized linear models with negative binomial distributions, adjusted for age, sex, and area of residence. **Results:** OBC-21 scores were positively associated with GAD-7 (R = 0.469, *p* < 0.001) and PHQ-9 (R = 0.432, *p* < 0.001). In adjusted models, OBC-21 remained significantly associated with anxiety symptoms (IRR = 1.0292, 95% CI: 1.0187–1.0399, *p* < 0.001) and depressive symptoms (IRR = 1.0293, 95% CI: 1.0187–1.0400, *p* < 0.001). Male sex was associated with lower anxiety scores, while age and area of residence were not significant. GAD-7 and PHQ-9 scores were strongly correlated. **Conclusions:** In this sample of adults attending private dental practices, a higher frequency of oral behaviors was associated with increased anxiety and depression symptoms, independently of age, sex, and area of residence. These findings support the clinical relevance of assessing oral behaviors as part of a biopsychosocial evaluation in dental practice.

## 1. Introduction

Oral behaviors represent a broad and relatively neutral term referring to repetitive, postural, or contact-related activities involving the stomatognathic system, beyond its basic physiological functions such as mastication, swallowing, and speech. In the present study, the term “oral behaviors” is used as the main terminology because the Oral Behaviors Checklist (OBC-21) assesses a broad range of self-reported behaviors rather than a single clinical diagnosis. “Oral overuse behaviors” refers more specifically to behaviors that may increase mechanical or functional loading of the masticatory muscles, teeth, or temporomandibular structures. “Oral parafunctions” is used as a related but more traditional clinical term for non-functional oral activities. By contrast, “bruxism” refers to a more specific masticatory muscle activity occurring during sleep or wakefulness, commonly characterized by clenching, grinding, bracing, or thrusting, and should not be considered synonymous with the full spectrum of oral behaviors assessed by the OBC-21 [1,2]. These behaviors can be assessed using the Oral Behaviors Checklist (OBC), a self-report instrument developed to identify and quantify oral behaviors and oral overuse activities [1]. Interest in this behavioral dimension has increased in recent years, as oral behaviors are considered relevant in the context of myofascial pain, temporomandibular disorders (TMD), and other manifestations involving overload of the masticatory system [2,3]. From a clinical perspective, oral behaviors are important not only through their frequency, but also through the mechanical and functional load they may impose on oro-facial structures. Repetitive episodes of clenching, grinding, or prolonged mandibular postures may contribute to the onset or persistence of temporomandibular symptoms, particularly in the presence of other predisposing or perpetuating factors [2,4]. In general dental populations, the prevalence of these behaviors is high, supporting their assessment not only in specialized TMD samples but also in routine dental settings [3]. Recent data from Romanian adults indicate that oral behaviors are also frequent in the local population, with a predominance of postural and involuntary activities and age-related variations [5].

In parallel, the role of psychological factors in the occurrence and maintenance of oral behaviors has been increasingly emphasized. Contemporary models of bruxism, oral parafunctions, and TMD place stress, anxiety, and depression within a biopsychosocial framework, in which emotional and behavioral responses may influence masticatory system activity [2,6]. A seminal analysis by Manfredini and Lobbezoo showed that available evidence supports an association between bruxism and factors such as anxiety, stress sensitivity, and depression, although the magnitude and consistency of these relationships depend on the type of bruxism and the diagnostic methods used [6]. More recent systematic reviews and meta-analyses have confirmed that psychological factors, particularly anxiety, depression, and stress, are significantly associated with TMD, despite important methodological heterogeneity [7,8,9].

Regarding the specific relationship between oral behaviors and emotional symptomatology, existing studies indicate a significant but still incompletely clarified association. Xu et al. [10] showed, in patients with TMD, that OBC scores were significantly associated with anxiety and depression, assessed using GAD-7 and PHQ-9, suggesting that individuals with higher oral behavioral load tend to present more pronounced emotional symptoms. Similar findings have been reported in other populations, including young adults and general dental patients, where oral behaviors were associated with psychological distress, personality traits, coping styles, or anxiety symptoms [3,11]. These findings support the idea that increased frequency of oral behaviors may reflect a broader behavioral expression of psychological vulnerability.

However, the current literature presents several limitations. Many studies have been conducted in specialized TMD samples or in specific populations, such as students or patients recruited from university clinics, which may limit the generalizability of the findings [3,10,11]. In addition, a large body of research has addressed the relationship between TMD and psychological factors more broadly, without specifically analyzing oral behaviors as a distinct behavioral variable [7,8,9]. In the Romanian context, available data on oral behaviors in adults remain limited and have focused mainly on prevalence and descriptive profiles rather than on their relationship with anxiety and depressive symptomatology [5]. Therefore, investigating this association in an adult sample recruited from dental practice, using standardized instruments for both behavioral and emotional dimensions, remains justified. The aim of the present study was to evaluate the association between self-reported oral behaviors, assessed using the OBC-21, and symptoms of anxiety and depression, assessed using GAD-7 and PHQ-9, in adults recruited from private dental practices in Romania. A secondary objective was to examine whether these associations remained present after adjustment for age, sex, and area of residence.

## 2. Materials and Methods

### 2.1. Study Design

This study had an observational, cross-sectional, analytical, and multicenter design and was conducted using the final version of a database developed within doctoral research. The present analysis was conducted on the final doctoral research dataset, which had been previously used in two related studies. The first study described the prevalence and patterns of oral behaviors in Romanian adults [5]. The second was a methodological study that used the psychosocial and behavioral components of the same dataset, namely OBC-21, GAD-7, and PHQ-9 scores, to develop and internally evaluate an interpretable artificial intelligence-based composite screening score [12]. In contrast, the present study addresses a distinct analytical objective: evaluating the association between self-reported oral behaviors and symptoms of anxiety and depression using correlation analysis and adjusted regression models.

No interventions were performed, and all data were obtained using self-administered psychometric instruments. All variables were assessed at a single time point, without longitudinal follow-up. The analysis included both descriptive and inferential components, examining the relationships between OBC-21, GAD-7, and PHQ-9 scores using bivariate and multivariable approaches. The multicenter design resulted from recruitment across multiple private dental practices within the same data collection network.

### 2.2. Participants

Participants were drawn from the final database used in the doctoral research. Between October 2024 and March 2025, 483 patients were assessed for eligibility in participating private dental practices. Of these, 23 (4.8%) were excluded: 7 (1.4%) due to incomplete data, 1 (0.2%) due to a known severe psychiatric disorder, 12 (2.5%) due to acute dental or oro-facial conditions, and 3 (0.6%) due to inability to comply with study procedures. No overlap between exclusion criteria was observed. The final sample included 460 participants.

Inclusion criteria were age ≥ 18 years, attendance at a participating dental practice during the study period, ability to complete questionnaires in Romanian, and provision of written informed consent. Exclusion criteria included incomplete data, severe psychiatric disorders, acute oro-facial conditions (e.g., acute pain, abscess, severe inflammation, recent trauma, or immediate postoperative status), and inability to comply with study procedures. Participants were consecutively recruited. This recruitment strategy generated a clinical convenience sample of adults attending private dental practices and was not designed to obtain a nationally representative sample of the Romanian adult population. Therefore, the demographic structure of the sample should be interpreted in relation to this recruitment setting. No formal screening or diagnostic assessment for temporomandibular disorders was performed. Participants with acute oro-facial conditions were excluded, but current or previous TMD diagnosis, TMD-related symptoms, or chronic oro-facial pain were not recorded and were not used as exclusion criteria.

The sample predominantly included women and urban residents, with the highest proportion in the 20–29 age group. The dataset was identical to that used in previous studies [5,12], with different analytical objectives.

### 2.3. Instruments

Three self-administered psychometric instruments were used: the Oral Behaviors Checklist (OBC-21), Generalized Anxiety Disorder-7 (GAD-7), and Patient Health Questionnaire-9 (PHQ-9) in accordance with the study objectives and the biopsychosocial orientation of the research [1,13,14].

The OBC-21 was used to assess the frequency of self-reported oral behaviors and was included as the main explanatory variable in the association analyses. GAD-7 and PHQ-9 were used as dependent variables to analyze the relationship between oral behaviors and anxiety and depressive symptoms.

The Romanian version of the OBC-21 used in the present study was not a formally validated Romanian version, but a linguistically adapted version developed for the research protocol. The original OBC-21 was translated into Romanian by two bilingual experts with expertise in dentistry and psychology [5]. The two translations were compared, discrepancies were resolved by consensus, and the final version was reviewed for conceptual and linguistic equivalence. Minor wording adjustments were made after pilot testing in a small group of Romanian-speaking adults to ensure item clarity and comprehensibility. No substantial difficulties in item comprehension were reported during pilot testing. However, a full psychometric validation of the Romanian OBC-21, including test–retest reliability, structural validity, and cross-cultural measurement invariance, was not performed. The internal consistency of the linguistically adapted Romanian OBC-21 was evaluated in the present sample using Cronbach’s alpha. This analysis was performed as a sample-specific reliability estimate and was not intended to represent a full psychometric validation of the Romanian version.

### 2.4. Data Collection

Data were collected between October 2024 and March 2025 using printed or digital questionnaires administered under standardized conditions in private dental practices. The present analysis used the final exported dataset (*n* = 460).

Participants were informed about the study purpose, voluntary participation, and data confidentiality. No additional procedures were introduced beyond the general research protocol.

### 2.5. Statistical Analysis

Statistical analyses were performed using IBM SPSS Statistics for Windows, Version 25.0 (IBM Corp., Armonk, NY, USA), with graphical outputs generated in Microsoft Excel and Microsoft Word for Microsoft 365 (Microsoft Corp., Redmond, WA, USA). Data distribution was assessed using the Shapiro–Wilk test. Quantitative variables were described using means and standard deviations or medians and interquartile ranges, while categorical variables were summarized using frequencies and percentages.

Given the non-normal distribution of psychometric scores, bivariate relationships between the investigated quantitative variables were analyzed using Spearman correlation coefficients. Generalized linear models were used to assess the relationship between OBC-21 and GAD-7 and PHQ-9 scores. Because GAD-7 and PHQ-9 are non-negative discrete symptom scores, Poisson regression was first considered as the standard count-data model. However, the Poisson assumption of equidispersion was evaluated and not supported, as the variance exceeded the mean for both outcomes and Poisson models showed evidence of overdispersion. Overdispersion was assessed using the Pearson χ^2^/degrees of freedom ratio. The frequency of zero scores was also examined to evaluate whether zero-inflation was a dominant feature of the data. Based on the presence of overdispersion and the limited proportion of zero values, negative binomial regression with a log link function was selected as the primary modeling approach. Separate models were constructed for each outcome. The OBC-21 score was included as the main variable of interest, while GAD-7 and PHQ-9 scores were analyzed as outcome variables. Age, sex, and area of residence were included as adjustment variables. The set of adjustment variables was limited to the demographic variables available in the dataset. Clinical, psychosocial, and socioeconomic variables such as TMD diagnosis, oro-facial pain severity, sleep disorders, perceived stress level, psychiatric medication use, educational or socioeconomic status, and bruxism diagnosis were not collected and therefore could not be included in the adjusted models.

Results were expressed as incidence rate ratios (IRR) with 95% confidence intervals. Statistical significance was set at α = 0.05. As an exploratory internal check, the internal consistency of the Romanian OBC-21 version in the present sample was assessed using Cronbach’s alpha, provided that all individual OBC-21 item responses were available.

### 2.6. Ethical Considerations

The study followed ethical principles for observational research involving human participants. Participation was voluntary and based on informed consent. Data were collected anonymously, without direct personal identifiers, and processed in accordance with data protection regulations.

The study did not involve additional clinical interventions or risks beyond routine dental care. It was conducted in accordance with the Declaration of Helsinki and approved by the Research Ethics Committee of the Faculty of Medicine and Pharmacy, University of Oradea (Decision no. CEFMF/2, 30 October 2023).

## 3. Results

### 3.1. Sample Characteristics and Distribution of OBC-21, GAD-7, and PHQ-9 Scores

The sample characteristics are presented in Table 1. The analyzed sample in the present study consisted of 460 participants. In terms of age distribution, the largest proportion was represented by the 20–29 age group (52%), followed by the 30–39 (22.4%) and 40–49 (11.3%) age groups. Participants were predominantly female (78.9%) and from urban areas (71.1%).

Regarding psychometric scores, the mean OBC-21 score was 22.51 ± 10.36 points, with a median of 21 and an interquartile range of 15–29, with observed values ranging from 0 to 60. For GAD-7, the mean was 7.63 ± 5.24 points, the median was 7, and the interquartile range was 4–11, with scores ranging from 0 to 21. In the case of PHQ-9, the mean was 7.24 ± 5.20 points, the median was 6, and the interquartile range was 3–10, with values ranging from 0 to 27.

Testing the distribution of quantitative variables using the Shapiro–Wilk test indicated significant deviations from normal distribution for all three investigated scores, which justified the use of non-parametric methods for correlation analysis and generalized linear models appropriate to the data distribution in the inferential stages of the study.

Overall, the analyzed sample exhibited sufficient variability in OBC-21, GAD-7, and PHQ-9 scores to allow the exploration of relationships between oral behaviors and anxiety and depressive symptomatology in subsequent analyses.

### 3.2. Internal Consistency of the Romanian OBC-21 Version

The internal consistency of the linguistically adapted Romanian OBC-21 was assessed in the present sample. Cronbach’s alpha for the total OBC-21 score was 0.825, indicating good internal consistency. The total OBC-21 score showed adequate coherence across items in this sample. However, this result should be interpreted as a sample-specific reliability estimate and not as evidence of full psychometric validation of the Romanian version.

### 3.3. Descriptive Profile of Symptoms Assessed by GAD-7 and PHQ-9

The distribution of responses to the GAD-7 and PHQ-9 questionnaire items is presented in Table 2 and Table 3. The descriptive analysis showed a predominant concentration of responses in the categories “not at all” and “several days”, suggesting a predominance of mild to moderate anxiety and depressive symptomatology in the analyzed sample.

In the case of anxiety symptoms, the most frequently reported manifestations were feeling easily annoyed or irritable, reported as occurring “several days” by 56.1% of participants, feeling nervous, anxious, or on edge, reported as occurring “several days” by 51.3% of participants, as well as excessive worrying about different things, present “several days” in 46.1% of respondents. Difficulties in relaxing and excessive restlessness also showed a high occasional frequency, being reported as occurring “several days” by 43.3% and 41.3% of participants, respectively. In contrast, the least frequent symptom was the persistent fear that something awful might happen, which was absent in 48.9% of participants (Table 2).

Although most anxiety symptoms showed a distribution concentrated in the “several days” category, some manifestations were also reported with higher frequency. Thus, 20.7% of participants indicated that feeling nervous, anxious, or on edge was present “more than half the days”, and 13.9% reported it “nearly every day”. In addition, difficulties in relaxing were reported “nearly every day” by 11.5% of participants, and irritability by 10.7%, suggesting the presence of a subgroup with more intense anxiety symptomatology (Table 2).

Regarding depressive symptomatology, the most frequently reported manifestation was feeling tired or having little energy, present “several days” in 46.3% of participants, “more than half the days” in 25.4%, and “nearly every day” in 17.6%. Other commonly reported symptoms included reduced interest or pleasure in activities, present “several days” in 48.5% of respondents, feeling down, depressed, or hopeless, reported “several days” in 45.4%, as well as sleep difficulties, present “several days” in 40.9% of participants. Changes in appetite also showed a relevant frequency, being reported “several days” by 38.3% of respondents (Table 3).

More severe depressive symptoms were reported less frequently. Negative feelings about oneself were absent in 60% of participants, concentration difficulties in 54.6%, and psychomotor retardation in 66.3%. The lowest frequency was observed for the item referring to thoughts that one would be better off dead or of self-harm, which was absent in 88.5% of cases, present “several days” in 8.7%, “more than half the days” in 2%, and “nearly every day” in 0.9% of participants (Table 3).

Overall, the descriptive profile of the GAD-7 and PHQ-9 items suggests a predominance of anxiety and depressive manifestations reported occasionally, with a low frequency of severe symptoms. At the same time, the distribution of responses indicates the presence of more prominent symptoms, particularly in the domains of irritability, psychological tension, fatigue, and sleep disturbances, while severe depressive manifestations had a relatively low frequency in the analyzed sample.

### 3.4. Association Between Oral Behaviors and Symptoms of Anxiety and Depression: Correlation Analysis

**Figure 1:**
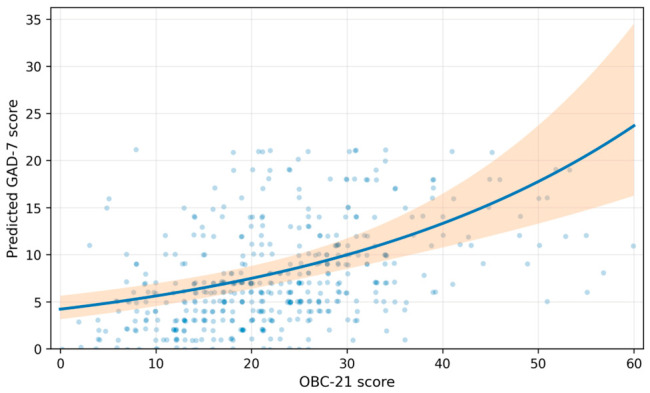
Adjusted association between OBC-21 scores and model-estimated GAD-7 scores. Estimates were derived from a negative binomial regression model adjusted for age, sex, and area of residence. Blue dots represent observed individual values, the blue line represents the adjusted model-estimated relationship, and the orange shaded area represents the 95% confidence interval. Estimates were derived from a negative binomial regression model adjusted for age, sex, and area of residence.

**Figure 2:**
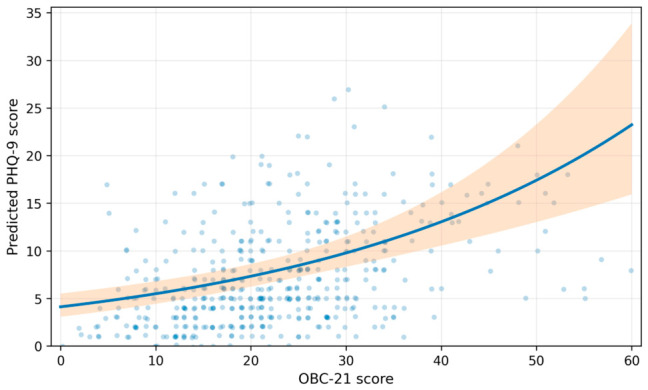
Adjusted association between OBC-21 scores and model-estimated PHQ-9 scores. Estimates were derived from a negative binomial regression model adjusted for age, sex, and area of residence. Blue dots represent observed individual values, the blue line represents the adjusted model-estimated relationship, and the orange shaded area represents the 95% confidence interval. Estimates were derived from a negative binomial regression model adjusted for age, sex, and area of residence.

The results of the correlation analysis between the OBC-21, GAD-7, and PHQ-9 scores are presented in Table 4. The adjusted relationships between OBC-21 and the predicted GAD-7 and PHQ-9 scores, based on the negative binomial regression models adjusted for age, sex, and area of residence, are illustrated in Figure 1 and Figure 2. The analysis was performed using Spearman’s correlation coefficient, given the non-parametric distribution of the investigated scores. The adjusted prediction plots showed a gradual increase in predicted GAD-7 and PHQ-9 scores across higher OBC-21 values, with 95% confidence intervals illustrating the uncertainty around the model-based estimates.

A positive, statistically significant, and moderate correlation was observed between the OBC-21 score and the GAD-7 score (R = 0.469; *p* < 0.001), indicating that participants with higher oral behavior scores tended to also present higher anxiety symptom scores.

Similarly, a positive, statistically significant, and moderate correlation was identified between the OBC-21 score and the PHQ-9 score (R = 0.432; *p* < 0.001), suggesting an association between a higher frequency of oral behaviors and greater severity of depressive symptoms (Table 4, Figure 1 and Figure 2).

### 3.5. Adjusted Association Between OBC-21 Score and GAD-7 Score

Before fitting the final negative binomial models, the assumptions of Poisson regression were evaluated for both outcome variables. For both GAD-7 and PHQ-9 scores, the variance was substantially higher than the mean, indicating overdispersion. In the Poisson models, the Pearson χ^2^/degrees of freedom ratio was 2.97 for GAD-7 and 3.03 for PHQ-9, confirming that the equidispersion assumption was not met. Zero scores were observed in 19 participants for GAD-7 (4.1%) and in 11 participants for PHQ-9 (2.4%), suggesting that zero-inflation was not a dominant feature of the outcome distributions. Therefore, negative binomial regression with a log link function was retained as the primary modeling approach.

The results of the generalized linear models assessing factors associated with GAD-7 scores are presented in Table 5. The multivariable analysis, performed using negative binomial regression with a log link function, indicated that the overall model was statistically significant.

In the model adjusted for age, sex, and area of residence, the OBC-21 score remained significantly associated with the GAD-7 score (IRR = 1.0292; 95% CI: 1.0187–1.0399; *p* < 0.001). This result indicates that each one-point increase in the OBC-21 score was associated with an estimated 2.9% increase in the GAD-7 score, while holding the other variables included in the model constant (Table 5).

Among the analyzed demographic variables, only sex showed a significant association with the GAD-7 score in the multivariable model. Thus, male sex was associated with lower GAD-7 scores compared to female sex (IRR = 0.755; 95% CI: 0.591–0.966; *p* = 0.026). In contrast, age and area of residence did not show statistically significant associations with GAD-7 scores in the adjusted model (Table 5).

Overall, the results of the multivariable model suggest that a higher frequency of oral behaviors is associated with higher levels of anxiety symptoms, even after controlling for the main demographic variables. At the same time, the data indicate the presence of a sex-related difference, with lower anxiety scores observed among men compared to women.

### 3.6. Adjusted Association Between OBC-21 Score and PHQ-9 Score

The results of the generalized linear models assessing factors associated with PHQ-9 scores are presented in Table 6. The multivariable analysis, performed using a negative binomial model with a log link function, indicated an overall statistically significant model (Likelihood Ratio χ^2^(8) = 44.450; *p* < 0.001), with an AIC value of 2775.495 and a McFadden pseudo-R^2^ of 0.177.

In the model adjusted for age, sex, and area of residence, the OBC-21 score remained significantly associated with the PHQ-9 score (IRR = 1.0293; 95% CI: 1.0187–1.0400; *p* < 0.001). This result indicates that each one-point increase in the OBC-21 score was associated with an estimated 2.9% increase in the PHQ-9 score, while holding the other variables included in the model constant (Table 6).

None of the analyzed demographic variables showed a statistically significant association with the PHQ-9 score in the multivariable model. Thus, after simultaneous adjustment for age, sex, and area of residence, the OBC-21 score remained the only significant associated with depressive symptomatology assessed by PHQ-9 (Table 6).

Overall, the results of the multivariable model suggest that a higher frequency of oral behaviors is associated with higher levels of depressive symptoms, independent of the main demographic characteristics included in the analysis.

## 4. Discussion

The present study showed that a higher frequency of oral behaviors, assessed using the OBC-21, was significantly associated with higher levels of anxiety and depression symptoms. This relationship was consistent across both correlation and adjusted models and remained significant after controlling for age, sex, and area of residence. Because of the cross-sectional design, the present findings do not allow conclusions regarding whether oral behaviors precede, follow, or interact bidirectionally with anxiety and depressive symptoms. Therefore, the observed relationships should be interpreted as associations within a shared biopsychosocial profile. GAD-7 and PHQ-9 scores were strongly correlated, while male sex was associated with lower anxiety scores, and age and area of residence were not significant.

Although the associations were statistically significant, the magnitude of the effect should be interpreted cautiously. The IRR of 1.029 indicates a modest increase in the expected GAD-7 and PHQ-9 scores for each one-point increase in OBC-21. However, because OBC-21 is a cumulative score, differences across clinically plausible ranges may be more informative than single-point changes. For example, a 14-point difference in OBC-21, corresponding approximately to the interquartile range observed in the present sample, would translate into an estimated 49% higher expected anxiety or depression symptom score, assuming other variables remain constant. Therefore, while the per-point effect is modest, the cumulative association across higher oral behavior loads may be clinically relevant. Nevertheless, the present study did not evaluate changes across established GAD-7 or PHQ-9 severity categories or determine minimal clinically important differences. Accordingly, the clinical meaningfulness of these associations should be interpreted with caution and investigated further in future studies. Therefore, the present findings should not be interpreted as demonstrating clinically meaningful changes in anxiety or depression severity at the individual-patient level but rather as indicating a statistically robust association that may become more relevant across larger differences in oral behavior load.

These results are consistent with previous studies reporting associations between oral behaviors and psychological factors within a biopsychosocial framework. Xu et al. [10] demonstrated similar relationships between OBC-21, GAD-7, and PHQ-9 in TMD patients, while other studies have linked oral behaviors with psychological distress, personality traits, and coping mechanisms across different populations [3,11,15,16,17]. Together, these findings suggest that oral behaviors and psychological symptoms may co-occur within a broader biopsychosocial profile, rather than being interpreted exclusively as isolated mechanical or local functional phenomena.

Several plausible mechanisms may help explain the observed association between oral behaviors and anxiety or depressive symptoms, although they cannot be confirmed by the present cross-sectional design. These mechanisms may be considered across four overlapping domains: stress physiology, neurobehavioral regulation, pain-processing mechanisms including central sensitization, and masticatory muscle hyperactivity related to oral parafunction. From a stress-physiology perspective, emotional distress may be accompanied by increased autonomic arousal and stress-related neuroendocrine responses, which can influence vigilance, muscle tone, and stress-related motor behaviors. In the stomatognathic system, this may be expressed through increased masticatory muscle activity, sustained tooth contact, clenching, mandibular bracing, or other non-functional oral behaviors [2,6]. These behaviors may increase mechanical loading of the masticatory muscles and temporomandibular structures and may contribute to symptom persistence in susceptible individuals [2].

Neurobehavioral and pain-processing pathways may also be relevant. Anxiety and depressive symptoms may influence attention to bodily sensations, symptom monitoring, pain perception, and the reporting of oral behaviors or oro-facial symptoms [7,8,9]. In this context, mechanisms such as altered pain modulation and central sensitization may be considered as possible explanatory pathways in TMD-related symptom persistence, but they were not directly assessed in the present study. Therefore, oral behaviors should not be interpreted only as local mechanical habits, but as part of a broader biopsychosocial pattern involving stress regulation, motor behavior, muscle activity, and pain-related processes. Future studies including clinical TMD diagnosis, pain assessment, electromyography, stress biomarkers, and measures of pain sensitivity are needed to test these mechanisms directly.

The opposite direction of the association should also be considered. Although the statistical models in the present study specified OBC-21 as the main explanatory variable and GAD-7 and PHQ-9 as outcome variables, this analytical structure should not be interpreted as evidence that oral behaviors precede emotional symptoms. Previous literature suggests that anxiety, depressive symptoms, and psychological distress may also contribute to increased oral behaviors through heightened arousal, increased muscle tension, altered attention to bodily sensations, and stress-related motor activity [2,6,10,17,18]. From this perspective, oral behaviors and emotional symptoms may influence each other within a bidirectional biopsychosocial pattern, rather than following a single causal pathway. Because the present study was cross-sectional, the temporal sequence between these variables could not be established.

This association is not limited to clinical populations. De Medeiros et al. [18] reported similar findings in dental students, and evidence indicates that the intensity of oral behaviors may show a dose–response relationship with psychological distress [19]. Cognitive factors may also influence self-reported behaviors, as suggested by van Selms et al. [20].

The relationship with depressive symptoms is also supported by the literature, although findings are more heterogeneous. Studies have shown associations between psychosocial impairment, TMD symptoms, and oral behaviors [4,10,21,22], with some evidence suggesting that pain may partially mediate these relationships [22].

The strong correlation between GAD-7 and PHQ-9 suggests that anxiety and depression symptoms should be interpreted within a shared psychological burden rather than as fully independent emotional outcomes. This is consistent with studies reporting high comorbidity and associations with TMD symptoms and reduced quality of life [23,24,25]. Accordingly, the two adjusted models presented in this study should be understood as parallel analyses of two closely related symptom dimensions, not as independent statistical evidence for two separate psychological constructs. In this context, higher OBC-21 scores may be interpreted as clinically relevant markers associated with a broader psychobehavioral profile, without implying a causal or directional relationship.

The observed association between sex and anxiety symptoms should be interpreted cautiously. Previous studies have reported higher psychosocial burden in women, including anxiety, depression, or TMD-related psychosocial impairment [21], although findings are not fully consistent across populations. In the present study, male sex was associated with lower GAD-7 scores in the adjusted model, whereas sex was not significantly associated with PHQ-9 scores after adjustment. Therefore, the sex-related pattern should be interpreted primarily in relation to anxiety symptoms rather than generalized to both anxiety and depression. Several explanations may be considered, including sex-related differences in emotional awareness, symptom reporting, willingness to disclose psychological distress, health-seeking behavior, and perceived quality of life [26,27]. Biological and psychosocial mechanisms, such as hormonal influences, social role expectations, and differential exposure to chronic stressors, may also contribute to sex-related differences in anxiety symptom burden. However, the present study did not assess gender roles, perceived stress, coping style, socioeconomic status, psychiatric history, or help-seeking behavior. Therefore, no conclusion can be drawn regarding the mechanism underlying this pattern in the Romanian population. This finding should be interpreted as a sample-specific association observed among adults attending private dental practices in Romania and should be examined in future studies using representative samples and variables specifically designed to assess sex- and gender-related mechanisms. The lack of significant effects for age and area of residence may be related to the demographic structure of the sample and to the use of a total OBC-21 score, as individual oral behaviors may show heterogeneous associations with clinical or psychosocial variables [28].

The demographic structure of the sample should be considered when interpreting the findings. The sample was predominantly female, urban, and relatively young, which limits the external validity of the results for the wider Romanian adult population. In addition, recruitment from private dental practices may have introduced selection bias, as individuals attending private dental care may differ from the general population in terms of access to dental services, health-seeking behavior, socioeconomic profile, and willingness to complete self-administered questionnaires. Therefore, the present sample cannot be considered representative of the general Romanian adult population or of all dental patients in Romania. Rather, the findings should be interpreted as applicable primarily to adults attending private dental practices within the recruitment network. Nevertheless, the sample characteristics are comparable to those reported in several clinical or academic studies involving oral behaviors and TMD-related psychosocial variables, where female predominance and clinical recruitment are frequently observed [10,24,25].

Overall, the findings are consistent with theoretical models describing the relationship between psychological distress and non-functional oral activity [2,29,30], as well as with evidence supporting associations between TMD and psychological factors [7,8,9]. Clinically, a high OBC-21 score, interpreted as a self-reported measure of oral behavioral load, may help identify patients in whom increased oral behaviors coexist with higher levels of anxiety and depressive symptoms. However, because TMD diagnosis and TMD-related symptoms were not assessed, these findings should not be interpreted as independent of TMD status [3,9,21,22].

Importantly, the present study differs from previous analyses conducted on the same dataset by focusing specifically on the association between oral behaviors and psychological symptomatology, rather than on model development, thereby providing a complementary perspective on the biopsychosocial relevance of oral behaviors.

### Limitations

Several limitations should be considered when interpreting the findings of the present study. First, the cross-sectional design does not allow conclusions regarding the temporal or causal direction of the association between oral behaviors and symptoms of anxiety and depression. Therefore, the observed relationships should be interpreted as associations rather than causal effects.

Second, the adjusted models included only the demographic variables available in the dataset, namely age, sex, and area of residence. Important clinical, psychosocial, and socioeconomic variables were not collected, including sleep disorders, perceived stress level, psychiatric medication use, bruxism diagnosis, educational level, income, occupation, and other indicators of socioeconomic status. The absence of socioeconomic data is particularly relevant because socioeconomic status may influence access to dental care, health-seeking behavior, psychological distress, anxiety, and depressive symptoms. The absence of these variables may have resulted in residual confounding. Consequently, the association between OBC-21 scores and GAD-7 or PHQ-9 scores should not be interpreted as independent of all clinically relevant factors.

A further important limitation concerns the absence of TMD screening or diagnostic assessment. Although participants were recruited from general private dental practices and acute oro-facial conditions were excluded, the study did not assess current or previous TMD diagnosis, TMD-related symptoms, chronic oro-facial pain, joint sounds, mandibular limitation, or pain-related disability. This is relevant because TMD and TMD-related symptoms are strongly associated with both oral behaviors and psychological distress. Therefore, undetected TMD symptoms may have contributed to the observed associations between OBC-21, GAD-7, and PHQ-9 scores. The present findings should therefore be interpreted as associations observed in a general dental-practice sample, not as associations independent of TMD status. Future studies should include standardized TMD screening or diagnostic assessment, such as DC/TMD-based evaluation, together with measures of oro-facial pain severity and pain-related disability.

Another limitation concerns the Romanian version of the OBC-21. The instrument used in the present study was linguistically adapted and pilot-tested for clarity and comprehensibility, but a full psychometric validation of the Romanian version was not performed. Although the linguistically adapted Romanian OBC-21 showed good internal consistency in the present sample (Cronbach’s alpha = 0.825), this result should be interpreted as a sample-specific reliability estimate rather than as evidence of full validation. Test–retest reliability, structural validity, construct validity, and measurement invariance were not evaluated. Therefore, the OBC-21 findings should be interpreted with caution, and future studies should perform a formal cross-cultural adaptation and psychometric validation of the Romanian OBC-21.

Another analytical limitation is that GAD-7 and PHQ-9 scores were strongly correlated. Therefore, the separate adjusted models for anxiety and depressive symptoms should not be interpreted as statistically independent analyses. Rather, they were used to examine whether the association with OBC-21 was consistent across two related dimensions of emotional symptomatology. Future studies may consider joint modeling approaches or composite psychological distress scores to better capture the shared variance between anxiety and depressive symptoms.

The sample was predominantly female, urban, and relatively young, and participants were recruited from private dental practices. This recruitment strategy may limit the generalizability of the findings to the wider Romanian adult population and may have introduced selection bias related to access to private dental care, health-seeking behavior, and willingness to complete self-administered questionnaires. Therefore, the findings should be interpreted primarily in relation to adults attending private dental practices within the recruitment network.

Finally, all variables were assessed using self-administered questionnaires, which may have introduced common method bias and shared method variance. Because oral behaviors, anxiety symptoms, and depressive symptoms were reported by the same participants using the same assessment format and at the same time point, the observed associations may have been partially influenced by common reporting tendencies, symptom awareness, negative affectivity, recall bias, or individual differences in response style. Although OBC-21, GAD-7, and PHQ-9 are widely used instruments, the exclusive reliance on self-reported measures limits the ability to distinguish between actual behavioral or clinical co-occurrence and associations amplified by the measurement method. Future studies should include clinical TMD assessment, oro-facial pain measures, sleep-related variables, perceived stress, medication use, socioeconomic indicators, validated bruxism assessment, objective measures of masticatory muscle activity, stress biomarkers, and pain-sensitivity assessment in order to better clarify the clinical significance and possible mechanisms underlying the observed associations.

## 5. Conclusions

In this clinical sample of adults recruited from private dental practices in Romania, higher OBC-21 scores were significantly associated with higher GAD-7 and PHQ-9 scores, and these associations remained significant after adjustment for age, sex, and area of residence. Because the sample was predominantly female and urban and was recruited from private dental practices, the findings should not be interpreted as representative of the wider Romanian adult population. Future studies using population-based or more demographically balanced samples are needed to confirm the external validity of these associations.

GAD-7 and PHQ-9 scores were strongly correlated, indicating substantial overlap between anxiety and depressive symptom burden. Therefore, the associations observed for anxiety and depressive symptoms should be interpreted in the context of this overlap rather than as fully independent findings. Among the demographic variables included in the models, only sex was significantly associated with anxiety symptoms, with lower GAD-7 scores observed in men. No demographic variable showed a significant association with PHQ-9 scores in the adjusted model.

Overall, these findings support the co-occurrence of oral behaviors and emotional symptoms within a shared biopsychosocial profile. However, due to the cross-sectional design, the results should be interpreted as associations rather than causal relationships.

Clinically, a higher frequency of self-reported oral behaviors may help flag dental patients who also report higher levels of anxiety and depressive symptoms. However, the clinical significance of the observed effect sizes requires further investigation. Longitudinal studies are needed to clarify the direction, temporal sequence, and clinical relevance of these relationships, incorporating additional variables such as oro-facial pain, sleep quality, perceived stress, bruxism status, and psychiatric history.

## Figures and Tables

**Table 1:** Characteristics of the analyzed sample.

Parameter	Value
Age (years)	number (%)
18	8 (1.7%)
20–29	239 (52%)
30–39	103 (22.4%)
40–49	52 (11.3%)
50–59	33 (7.2%)
60–69	18 (3.9%)
70–79	6 (1.3%)
≥80	1 (0.2%)
Sex—female	363 (78.9%)
Urban area of residence	327 (71.1%)
Psychometric scores	Mean ± SD, Median (IQR), Min–Max
OBC-21 (*p* < 0.001 *)	22.51 ± 10.36, 21 (15–29), 0–60
GAD-7 (*p* < 0.001 *)	7.63 ± 5.24, 7 (4–11), 0–21
PHQ-9 (*p* < 0.001 *)	7.24 ± 5.2, 6 (3–10), 0–27

Note: SD = standard deviation; IQR = interquartile range; * Shapiro–Wilk test.

**Table 2:** Distribution of responses to GAD-7 items.

GAD—7 Items	Not at All *n* (%)	Several Days *n* (%)	More Than Half the Days *n* (%)	Nearly Every Day *n* (%)
GAD 1	65 (14.1)	236 (51.3)	95 (20.7)	64 (13.9)
GAD 2	152 (33.0)	216 (47.0)	50 (10.9)	42 (9.1)
GAD 3	108 (23.5)	212 (46.1)	83 (18.0)	57 (12.4)
GAD 4	127 (27.6)	199 (43.3)	81 (17.6)	53 (11.5)
GAD 5	154 (33.5)	190 (41.3)	70 (15.2)	46 (10.0)
GAD 6	77 (16.7)	258 (56.1)	76 (16.5)	49 (10.7)
GAD 7	225 (48.9)	152 (33.0)	46 (10.0)	37 (8.0)

Note: values are expressed as absolute frequencies and percentages.

**Table 3:** Distribution of responses to PHQ-9 items.

PHQ—9 Items	Not at All *n* (%)	Several Days *n* (%)	More Than Half the Days *n* (%)	Nearly Every Day *n* (%)
PHQ 1	159 (34.6)	223 (48.5)	58 (12.6)	20 (4.3)
PHQ 2	165 (35.9)	209 (45.4)	66 (14.3)	20 (4.3)
PHQ 3	131 (28.5)	188 (40.9)	80 (17.4)	61 (13.3)
PHQ 4	49 (10.7)	213 (46.3)	117 (25.4)	81 (17.6)
PHQ 5	160 (34.8)	176 (38.3)	78 (17.0)	46 (10.0)
PHQ 6	276 (60.0)	128 (27.8)	35 (7.6)	21 (4.6)
PHQ 7	251 (54.6)	148 (32.2)	37 (8.0)	24 (5.2)
PHQ 8	305 (66.3)	111 (24.1)	29 (6.3)	15 (3.3)
PHQ 9	407 (88.5)	40 (8.7)	9 (2.0)	4 (0.9)

Note: values are expressed as absolute frequencies and percentages.

**Table 4:** Correlation matrix for the analyzed psychometric scores.

Correlation *	OBC-21	GAD-7	PHQ-9
OBC-21	-	R = 0.469, *p* < 0.001	R = 0.432, *p* < 0.001
GAD-7	R = 0.469, *p* < 0.001	-	R = 0.759, *p* < 0.001
PHQ-9	R = 0.432, *p* < 0.001	R = 0.759, *p* < 0.001	-

Note: * Spearman’s rho Correlation Coefficient.

**Table 5:** Univariate and multivariable generalized linear models assessing factors associated with GAD-7 scores.

Parameter	Univariate		Multivariable *	
	IRR (95% CI)	*p*	IRR (95% CI)	*p*
Age—years				
18–29 (Ref.)	-	-	-	-
30–39	0.833 (0.652–1.065)	0.144	0.946 (0.736–1.216)	0.665
40–49	0.833 (0.606–1.146)	0.263	0.893 (0.647–1.232)	0.490
50–59	0.783 (0.530–1.155)	0.217	0.908 (0.610–1.350)	0.633
60–69	0.641 (0.381–1.079)	0.094	0.919 (0.537–1.572)	0.757
≥70	0.595 (0.262–1.354)	0.216	1.064 (0.455–2.489)	0.886
Sex (male)	0.752 (0.591–0.957)	0.020	0.755 (0.591–0.966)	0.026
Area of residence (urban)	1.066 (0.860–1.322)	0.559	1.043 (0.839–1.297)	0.704
OBC-21	1.030 (1.020–1.040)	<0.001	1.0292 (1.0187–1.0399)	<0.001

Note: * Multivariable model adjusted for age, sex, and area of residence; IRR = incidence rate ratio; 95% CI = 95% confidence interval; Ref. = reference category; For the multivariable model: χ^2^(451) = 172.739; likelihood ratio test χ^2^(8) = 43.062, *p* < 0.001; McFadden pseudo-R^2^ = 0.165. Model effects: age, *p* = 0.979; sex, *p* = 0.026; area of residence, *p* = 0.704; OBC, *p* < 0.001.

**Table 6:** Univariate and multivariable generalized linear models assessing factors associated with PHQ-9 scores.

Parameter	Univariate		Multivariable *	
	IRR (95% CI)	*p*	IRR (95% CI)	*p*
Age—years				
18–29 (Ref.)	-	-	-	-
30–39	0.741 (0.579–0.948)	0.017	0.803 (0.625–1.032)	0.803
40–49	0.735 (0.533–1.014)	0.061	0.793 (0.573–1.097)	0.161
50–59	0.751 (0.508–1.111)	0.152	0.868 (0.583–1.291)	0.484
60–69	0.591 (0.350–0.999)	0.049	0.819 (0.478–1.404)	0.468
≥70	0.829 (0.371–1.853)	0.829	1.437 (0.626–3.298)	0.392
Sex (male)	0.824 (0.648–1.049)	0.115	0.822 (0.643–1.050)	0.117
Area of residence (urban)	1.069 (0.862–1.326)	0.546	1.036 (0.833–1.289)	0.752
OBC-21	1.030 (1.020–1.040)	<0.001	1.0293 (1.0187–1.0400)	<0.001

Note: * Multivariable model adjusted for age, sex, and area of residence; IRR = incidence rate ratio; 95% CI = 95% confidence interval; Ref. = reference category. For the multivariable model: χ^2^(451) = 180.393; likelihood ratio test χ^2^(8) = 44.450, *p* < 0.001; McFadden pseudo-R^2^ = 0.177. Model effects: age, *p* = 0.348; sex, *p* = 0.117; area of residence, *p* = 0.752; OBC, *p* < 0.001.

## Data Availability

The original contributions presented in this study are included in the article. Further inquiries can be directed to the corresponding authors on reasonable request.

## Abbreviations

The following abbreviations are used in this manuscript:

OBC-21	Oral Behavior Checklist
GAD-7	Generalized Anxiety Disorder-7
PHQ-9	Patient Health Questionnaire-9
TMDs	Temporomandibular Disorders
